# Editorial: Substance use disorder: above and beyond addiction

**DOI:** 10.3389/fphar.2024.1426351

**Published:** 2024-05-15

**Authors:** Jaya Kumar, Wael Mohamed, Dasiel Oscar Borroto-Escuela, Muthuraju Sangu, Rusdi Rashid, Mohd Fadzli Mohamad Isa, Prem Kumar Shanmugam

**Affiliations:** ^1^ Department of Physiology, Faculty of Medicine, Universiti Kebangsaan Malaysia, Kuala Lumpur, Malaysia; ^2^ Basic Medical Science Department, Kulliyyah of Medicine, International Islamic University Malaysia, Kuantan, Malaysia; ^3^ Department of Neuroscience, Karolinska Institutet (KI), Stockholm, Sweden; ^4^ Receptomics and Brain Disorders Lab, Department of Human Physiology, Physical Education, and Sport, Faculty of Medicine, University of Malaga, Málaga, Spain; ^5^ Department of Radiology, Houston Methodist Academic Institute (HMAI), Houston, TX, United States; ^6^ Department of Psychological Medicine, Faculty of Medicine, University of Malaya, Kuala Lumpur, Malaysia; ^7^ Department of Psychiatry and Mental Health, Hospital Kuala Lumpur, Kuala Lumpur, Malaysia; ^8^ Psychology, Manchester Metropolitan University, Kota Kinabalu, Malaysia

**Keywords:** substance use disorder, addiction, drug dependence, drug addicion, alcohol, stimulant, substance

Substance use disorder (SUD) has been, for an extended period, seen as a form of addiction and a chronic brain illness. The Research Topic *“Substance use disorder: above and beyond addiction”* adopts a wider perspective, delving into the multi-dimensional aspects of SUD and its numerous driving factors that result in its development, maintenance, and relapse, in addition to the management of the disease. We received a substantial number of submissions for this Research Topic, and 31 articles were finally published.

Alcohol use disorder (AUD) affects millions of individuals worldwide. Reflecting its widespread impact, we received multiple submissions addressing this critical area of addiction. Beaurepaire and Rolland reported that high-dose baclofen significantly reduced alcohol consumption and alcohol cravings and increased the number of subjects with minimal or no-risk alcohol consumption, based on data from a French program between 2014 and 2017. Building upon this, Driver et al. illuminated that web-based educational tools can be effective in improving the public’s understanding of polygenic risk scores for AUD, as demonstrated by a randomized controlled trial involving 325 college students. Meanwhile, Fortinguerra et al. found that medications to treat AUD in Italy accounted for only 0.018% of all medications. Almost half of these medications were dispensed through public healthcare facilities, 23.5% through community pharmacies, and 23.3% through private purchases. Expanding our understanding to regional perspectives, Mulualem Belay et al. systematic review and meta-analysis, revealed that in East Asian adults, Korea had the highest 1-year and lifetime pooled prevalence of AUD, with being male and smoking recognized as the strongest risk factors for developing AUD. In line with these findings, Chen et al. investigated how male patients with alcoholic pancreatitis post-recovery perceive withdrawal and life management, covering five themes such as staying sober, change in role, status of illness, life management, and family influence. The findings from this study would help develop targeted interventions for these patients to maintain a healthy lifestyle. Truque et al. added another layer by examining the cognitive ramifications of binge drinking among college students, revealing associations between binge drinking and impaired verbal memory and reduced use of memory strategies when recalling information. Additionally, binge drinkers also made more mistakes in memory tests.

Opioid use, be it illicit or prescribed use, is a significant public health concern, due to its high addiction liability, and other adverse effects. In a pilot study, Jovin et al. unveiled a concerning trend, where one-third of the 64 opioid-dependent patients in Serbia were using synthetic cannabinoids, and most of them were using other substances such as alcohol, marijuana, and multiple psychoactive substances. Furthermore, Goulete et al. shed light on the unique demographic profile of veterans navigating the dual healthcare system. They found that veterans who receive opioid prescriptions at the Veterans Administration (VA) healthcare system and private healthcare (dual system) are more likely to be female, younger than mono users, and less likely to be white. Dual-system users face a heightened risk of receiving excessive opioid prescriptions, underscoring the need for improved coordination between both healthcare systems. Meanwhile, Yuen et al. explored a novel therapeutic avenue, such as the potential of deep brain stimulation to mitigate the rewarding effects of oxycodone and associated respiratory changes. Their findings merit further exploration as a potential therapy for managing the impact of oxycodone on the reward center and respiration. Bringing additional depth in understanding opioid effects, Gao et al. reported that paternal chronic heroin self-administration may affect analgesic responses to heroin among male offspring without impacting attention behavior or reinforcing the effects of cocaine. Furthermore, Kaplan and Thompson, in their review, elucidated how neuroplasticity occurring in the extended amygdala during opioid withdrawal contributes to the emergence of negative emotions and cravings.

Like other drugs of abuse, cocaine has profound impact on individual wellbeing. In a study by Merritt et al., rats were trained to self-administer cocaine and categorized them as “high cue” or “low cue” responders based on their lever pressing response to previously linked cocaine cues. They observed a significant difference in the expression of 309 genes in the medial prefrontal cortex between these two groups of rats, with particular emphasis on the gene encoding the serotonin 5-HT2C receptor (5HT2CR), which exhibited downregulation in the “high cue” group. This suggests that 5HT2CR could be a potential target in pharmacotherapy for cocaine use disorder (CUD). Shedding light on the human experience, Maahs et al. noted that individuals with CUD hesitate to seek treatment for their drug use problem due to stigma, privacy concerns, being labelled as drug seekers, lack of time, and treatment affordability. According to the survey findings, patients also underscored the importance of safe and effective therapy in alleviating withdrawal symptoms and cravings. Further insights into the neurological impact of CUD were provided by Schinz et al., who reported that individuals with CUD had significantly thinner cortex in brain areas involved in memory, planning, and attention. Furthermore, the duration of cocaine use was associated with accelerated brain aging.

COVID-19 pandemic had significant impact on mental health worldwide. In a study by Mielau et al., findings revealed that the use of cannabis during the early stages of the COVID-19 pandemic (1 month after its onset) was nearly identical to usage levels prior to the pandemic. Transitioning to neurological insights, Li et al. uncovered significant hyperconnectivity between the left lateral amygdala and the left inferior/middle occipital gyrus, as well as the left middle/superior temporal gyrus, which gradually normalizes after long-term abstinence in individuals abstaining from methamphetamine (METH). Extending these insights, Jiang et al. research in the Chinese Han population, identified gene prokineticin 2 (PROK2) as important for susceptibility to developing methamphetamine use disorder and cravings.

In an intriguing research work, Sanchez and Amaro discovered that increased exposure to traumatic events and emotional dysregulation are correlated with cravings for substances of abuse, suggesting that interventions targeting emotional regulation and mindfulness could be beneficial in managing substance use disorder (SUD) in racial and ethnic minority women. Echoing this sentiment, Pereira et al. highlighted the potential of the Interpersonal Theory of Anxiety Management in People with Substance Use Disorders (ITASUD) to enhance treatment outcomes by assisting SUD patients in managing their anxiety levels. Transitioning to innovative approaches in addressing substance use Research Topic, Gray et al. aimed to evaluate the effectiveness of a novel approach called iDECIDE (Drug Education Curriculum: Intervention, Diversion, and Empowerment), a skill-based program developed as an alternative to punishment for addressing students with substance use problems. In alignment with the theme of integrated treatment approaches, Khalid et al. revealed that the community reinforcement approach (CRA) is an effective tool to be integrated with other treatment models for treating SUD in Pakistan. Further exploring the psychological aspects of substance use, Maftei and Opariuc-Dan found that a low level of happiness is associated with self-criticism, narcissistic perfectionism, substance use, and internet addiction. Furthermore, a high level of perfectionism is associated with health-risk behaviors, subsequently leading to unhappiness. Building upon the understanding of substance-related harms, through a retrospective study, Liu et al., investigated the characteristics of individuals hospitalized for acute poisoning between 2017 and 2022, it was revealed that drugs of abuse were the most frequently reported poisons, followed by pesticides, carbon monoxide, and alcohol. Suicide emerged as the primary cause of poisoning, with underlying medical conditions and female gender being identified as risk factors for suicide attempts or suicide. Proposing a paradigm shift in addiction therapy, Unterrainer introduced a novel approach to addiction therapy by relooking at addiction as an existential neurosis, hence exploring hope, meaning, and purpose in recovery. Adding to the discourse on holistic approaches to addiction treatment, Tyler et al. discovered that high-intensity interval workouts markedly enhanced the binding of dopamine receptor 2 (DR2) in the nucleus accumbens shell, in comparison to the sedentary group. The researchers also reported male rats having higher D2R binding. The findings offer insight on how HIIT could potentially assist in addiction treatment. Furthermore, Amanollahi et al. found patients suffering from AUD are more likely to develop liver and kidney-related complications, whereas heart problems are more common in opioid and simulant-using patients. Men were more likely to develop liver and kidney problems as well as mortality, and psychoactive substance use appears to offer some protection against cardiovascular-related complications and mortality in this group of patients.

The use of e-cigarettes has become increasingly prevalent in recent years. Elucidating this aspect further, Liu et al. reported that among Taiwanese high school students, 9.3% used e-cigarettes, and favorable reactions of close friends towards e-cigarettes, tobacco smoking, and use of other substances were risk factors for e-cigarette use. In addition to this, Zhang and Wen, in their narrative review, explored the effects of electronic nicotine delivery systems on oral health compared to conventional cigarettes. Transitioning to perceptions of harm and addiction, Atem et al. uncovered that adolescents who perceived tobacco products or hookah as less harmful were more prone to initiate their use at a young age, while those who viewed hookah as less addictive were less inclined to start using it at a younger age. These findings emphasized on the significance of tackling misconceptions and enhancing awareness among youth regarding the risks associated with tobacco and hookah use.

Behavioral addictions associated with technologies have garnered significant attention lately. In a notable study, Park et al. found that adults with internet gaming disorder and AUD are more vulnerable to stress due to their lower heart rate variability (HRV). This suggests that HRV could serve as a common transdiagnostic marker of addiction. Expanding on this notion, Zhao et al. uncovered a reciprocal relationship between meaning in life and smart phone addiction among Chinese college students and highlighted gender variations in the associations.

We hope that this Research Topic will shed insight on the multifaceted nature of SUD, extending beyond its traditional focus solely on brain illness or addiction. We anticipate that the articles published on this Research Topic would hold value for clinicians, researchers, and policymakers in the field of SUD. Consequently, this is expected to foster further development of comprehensive and holistic approaches to both understanding and managing SUD.

